# Clinical Predictors of Mood Disorders and Prevalence of Neuropsychiatric Symptoms in Patients with Systemic Lupus Erythematosus

**DOI:** 10.3390/jcm13185423

**Published:** 2024-09-13

**Authors:** María Recio-Barbero, Janire Cabezas-Garduño, Jimena Varona, Guillermo Ruiz-Irastorza, Igor Horrillo, J. Javier Meana, Borja Santos-Zorrozúa, Rafael Segarra

**Affiliations:** 1 BioBizkaia Health Research Institute, 48903 Barakaldo, Spain; maria.reciobarbero@bio-bizkaia.eus (M.R.-B.); janire.cabezasgarduno@osakidetza.eus (J.C.-G.); jimena.varonaperez@osakidetza.eus (J.V.); igor.horrillo@ehu.eus (I.H.); javier.meana@ehu.eus (J.J.M.); borja.santoszorrozua@bio-bizkaia.eus (B.S.-Z.); rafael.segarraechevarria@osakidetza.eus (R.S.); 2 University of the Basque Country, UPV/EHU, 48940 Leioa, Spain; 3 Department of Psychiatry, Cruces University Hospital, 48903 Barakaldo, Spain; 4 Autoimmune Disease Unit, Department of Internal Medicine, Cruces University Hospital, 48903 Barakaldo, Spain; 5 Centro de Investigación Biomédica en Red de Salud Mental, CIBERSAM ISCIII, 48940 Leioa, Spain

**Keywords:** mood disorders, depressive disorder, neuropsychiatric lupus, systemic lupus erythematosus, autoimmune disorders

## Abstract

**Background/Objectives:** We aimed to determine the prevalence and clinical correlations of mood disorders in a sample of systemic lupus erythematosus (SLE) patients. Hence, we hypothesized that the prevalence of mood disorders would be lower than reported in the literature and that patients would remain clinically stable and show less damage accrual despite low-dose corticosteroid prescription. **Methods:** In total, 92 SLE outpatients gave informed consent to participate in this cross-sectional study. Psychiatric and autoimmune clinical data were obtained, and a structured psychiatric interview was performed. The main clinical scales for the assessment of clinical symptomatology were included. To examine the potential relationships of presenting a mood disorder in SLE, clinical correlations and multivariate analyses were performed. **Results:** Mood disorders were the most prevalent disorder reported by SLE patients (16%), followed by adjustment disorders (5%). A significant proportion of patients presented psychosocial disturbances that did not meet the ICD-10 criteria for psychiatric diagnosis. According to the cut-off criterion for the Montgomery–Åsberg Depression Rating Scale (MADRS), up to 27% of the sample met the clinical criteria for depression. The multivariate analysis revealed a relationship between the presence of a mood disorder with total scores of the MADRS and the Young Mania Rating Scale (YMRS). **Conclusions:** The prevalence of mood disorders in patients with SLE was lower than previously reported. Although self-report clinical scales are useful for assessing clinical symptomatology, they should not be used in place of a comprehensive standardized interview conducted by a trained mental health specialist. Multidisciplinary teamwork is required for the early identification and therapeutic management of autoimmune patients with neuropsychiatric disorders.

## 1. Introduction

Systemic lupus erythematosus (SLE) is a chronic autoimmune disease with the potential to affect multiple organ systems. The etiology of SLE includes the interaction between genetic and environmental components, resulting in immune dysregulation and the breakdown of self-tolerance [1]. Humoral immunity plays a major role in the pathogenesis of SLE, with the production of a wide range of autoantibodies, some of them with a well-defined pathogenic activity, such as anti-DNA in lupus nephritis, anti-Ro in neonatal lupus, and antiphospholipid in thrombotic events [1]. However, the disease is much more complex, with the participation of cellular compartments of both the adaptive and innate immune response, which results in a broad spectrum of clinical manifestations [1]. Female gender strongly influences the pathogenesis, with a resultant female/male ratio of 10/1 [2]. The course of lupus includes periods of remission and flares, leading to chronic inflammation [3,4], which, if not treated promptly and adequately, can cause irreversible organ damage, thus reducing survival and the health-related quality of life [4,5,6].

SLE symptoms may also present with related neuropsychological disturbances. Indeed, patients suffering from autoimmune diseases such as multiple sclerosis and rheumatoid arthritis, in addition to SLE, have reported a higher incidence of neuropsychiatric disturbances, with prevalence rates ranging from 15% to 75% [7,8,9,10]. Nervous system disturbances are frequently reported in SLE [1] and may affect both the central nervous system (CNS) and the peripheral nervous system (PNS), resulting in a wide range of diffuse neurologic and neuropsychiatric conditions (e.g., headache, acute confusional state, seizures, psychosis, mood disorders, and cognitive dysfunction) [3].

While nervous system involvement in SLE remains one of the major causes of morbidity and mortality [5], its etiology and pathogenesis remain unclear [11]. In other words, SLE encompasses a wide spectrum of clinical features, among which physical and neuropsychiatric symptoms, together with psychosocial disturbances, appear to stand out [12]. Aiming to identify the most prevalent neuropsychiatric disorders in SLE patients, in 1999, the American College of Rheumatology (ACR) proposed a classification criterion for 19 CNS and PNS syndromes, collectively referred to as Neuropsychiatric SLE (NPSLE) [13]. Despite several efforts to improve the sensitivity and specificity of the SLE criteria, NPSLE diagnosis includes a miscellaneous category of nonspecific neurologic and psychiatric signs. While neurological manifestations have been always grouped together, the criteria for psychiatric disorders have been revised over the years, exhibiting variable prevalence rates [3,14,15].

In this regard, a recent meta-analysis concluded that a high proportion of SLE patients had depressive and anxiety symptoms, with a pooled prevalence of 35% and 25.8%, respectively [15]. However, most of the published studies addressing neuropsychiatric disorders in autoimmune disorders mostly rely on clinical screening tools [14], lacking a comprehensive standardized psychiatric assessment by qualified mental health specialists.

Given that psychiatric disorders have been reported to be among the leading disabling conditions globally [16,17], estimating the true prevalence of these disorders remains necessary to better understand their true impact on patients’ quality of life. The aim of this study was to assess the prevalence and clinical correlations of well-defined mood disorders in our cohort of SLE patients.

## 2. Materials and Methods

### 2.1. Study Sample

This study utilized a cross-sectional design to assess a sample of 92 patients with systemic lupus erythematosus (SLE) who were actively being followed up at the Autoimmune Diseases Unit at Cruces University Hospital, Spain, the Lupus–Cruces Cohort. This is a well-established cohort, as detailed elsewhere [18]. A cross-sectional study involves data collection at a single point in time, offering a snapshot of the study population at that moment. Unselected consecutive patients attending outpatient clinics between 2017 and 2022 were invited to participate, provided they met the inclusion and exclusion criteria. The inclusion criteria were an age of over 18 years old, a current diagnosis of SLE according to the revised criteria from the ACR/EULAR consensus for the classification of SLE [19], and signing the informed consent form. The exclusion criteria were a history of neurologic damage (including severe head injury, neurodegenerative, vascular or metabolic disorder, and neoplasia); the concurrence of severe or terminal somatic disease; and physical, sensory, or intellectual incapacity impeding the completion of the study protocol. No further criteria related to demographic or clinical characteristics were used, so that the group of study could be considered representative of the whole Lupus–Cruces Cohort. The study was approved by the Basque Ethics Committee (CEI-Euskadi PI2017029, last approval 30 August 2020).

### 2.2. Psychiatric Assessment

All the participants included in this study were screened by a trained psychiatric specialist using the Semi-Structured Clinical Interview for Axis I DSM-IV Disorders (SCID) [20]. In addition, each participant was screened for major psychiatric disorders according to the 10th Revision of the International Statistical Classification of Diseases and Related Health Problems (ICD-10) [21].

Complementarily, patients were assessed for a broad spectrum of psychiatric symptoms, including depressive and anxious symptomatology, the presence of suicidal ideation, and past traumatic experiences, concluding with the overall global clinical impression using widely utilized psychometric scales in the clinical setting. The scales used are detailed below.

Montgomery–Åsberg Depression Rating Scale (MADRS) [22]: The MADRS is a validated tool of ten items covering various aspects of depression, including affective symptoms (e.g., depressed mood, irritability), somatic symptoms (e.g., sleep disturbances, appetite changes), and cognitive symptoms (e.g., feelings of guilt, suicidal ideation). Each item is rated on a 7-point Likert scale ranging from 0 (no symptoms) to 6 (severe symptomatology). Total scores range from 0 to 60, with higher scores indicating more severe depression.

Hamilton Anxiety Rating Scale (HARS) [22]: The HARS is a clinician-administered widely used instrument for assessing the severity of anxiety. It comprises 14 items that evaluate both psychological (e.g., tension, fear) and somatic (e.g., restlessness, insomnia) manifestations of anxiety. Each item is rated on a 4-point Likert scale ranging from 0 (no symptoms) to 4 (severe symptoms). Total scores range from 0 to 56, with higher scores indicating greater anxiety severity.

Young Mania Rating Scale (YMRS) [23]: The YMRS is a widely used tool designed to assess the severity of manic symptoms in individuals with bipolar disorder. The scale consists of 11 items evaluating core manic symptoms, including elevated mood, increased motor activity, irritability, disruptive behavior, grandiosity, and decreased sleep. Items are rated on either a 4-point or 8-point Likert scale depending on the item. The total scores range from 0 to 60 (or higher depending on the scoring method), with higher scores indicating more severe manic symptoms.

Plutchik Suicide Risk Scale [24]: The Plutchik Suicide Risk Scale is a 15-item self-report measure designed to assess suicide risk. It evaluates various factors associated with suicidal behavior, such as suicidal ideation, previous suicide attempts, and hopelessness. It consists of 15 items that explore various factors related to suicidal ideation, behavior, and intent. Each item is scored as either 0 (no) or 1 (yes), resulting in a total score ranging from 0 to 15. Higher scores indicate an increased risk of suicide.

Traumatic Experiences Screening Questionnaire (ExpTra-S) [25]: The ExpTra-S is a self-report screening tool used to assess exposure to traumatic experiences in individuals. It consists of 18 items rated on a 4-point Likert scale (0 = never to 3 = almost always). The scale covers various types of child abuse (sexual, physical, psychological, and neglect) and includes an open-ended item for other traumatic events. The distress scale, also composed of 18 items rated on a 4-point Likert scale (1 = no distress to 4 = great distress), assesses the emotional impact of these experiences.

Global Clinical Impression Scale (CGI) [26]: The CGI is a clinician-rated instrument used to provide an overall assessment of a patient’s clinical status and severity of illness. It consists of two main components: CGI-S (severity) and CGI-I (improvement). The CGI-S is based on the clinician’s global impression, using a 7-point scale to rate symptom severity on a scale of 1 (normal) to 7 (extremely ill).

### 2.3. Clinical Assessment of Lupus Patients

The sociodemographic and clinical characteristics of the study group, including immunomodulatory treatments, were collected for this study. The Systemic Lupus Erythematosus Disease Activity Index (SLEDAI-2K) [27] was used to assess the SLE disease activity. This original SLEDAI was developed in 1985 and later modified to SLEDAI-2K including minor changes [27]. It consists of 24 items comprising 16 clinical (such as rash, arthritis, pleuritis, or psychosis) and 8 laboratory values (including elevated anti-DNA antibodies or hypocomplementemia). Each item has a specific score ranging from 1 to 8, so the global score has a possible maximum score of 105. SLEDAI-2K global scores up to 5 are considered mild activity, 6–12 moderate activity, and >12 severe activity.

Likewise, The Systemic Lupus International Collaborating Clinics/ACR Damage Index (SDI) was used to score the degree of cumulative irreversible damage caused by the disease, therapeutic agents, or concurrent conditions [28,29]. The SDI represents permanent damage, in contrast with SLEDAI-2K, which measures reversible activity. To be counted, items should be present for at least 6 months (with the exception of myocardial infarction and stroke) and need not be attributed to SLE. SDI items are grouped into 12 organ systems: ocular, neuropsychiatric, renal, pulmonary, cardiovascular, peripheral vascular, gastrointestinal, musculoskeletal, skin, endocrine (diabetes), gonadal dysfunction, and malignancy. Notably, the SDI can only be stable or increase over time, with a maximum possible score of 47 points. The SDI has been shown to be a major prognostic predictor in SLE patients [1].

### 2.4. Data Analysis

Descriptive statistics were calculated for the examined variables. Continuous variables are reported as the mean (standard deviation) for normally distributed data and otherwise as the median (interquartile range). Categorical variables are presented as frequency (percentage). To determine normality, the Shapiro–Wilks test was used. Comparisons between groups were made with Student’s *t*-test in the case of continuous variables following a normal distribution or with the nonparametric Mann–Whitney U test otherwise. The Chi-square test or Fisher’s exact test was used for categorical data.

To estimate the associations between the studied variables, Pearson’s or Spearman’s correlation coefficients were calculated. Multivariate logistic regression models were applied to identify the predictors of affective disorder in patients with SLE. For univariate analysis, clinical variables with *p* ≤ 0.15 were used in the final regression model. Collinearity between candidate variables was analyzed with the Spearman correlation and VIF (variance inflation factor) coefficients. The final model calibration was assessed with the Hosmer–Lemeshow goodness-of-fit test (*p* > 0.05).

Finally, the area under the ROC curve (AUC) was calculated for the assessment of model discrimination and diagnostic accuracy. For hypothesis testing, a 95% confidence interval was considered, setting the risk α of 0.05 as the limit of statistical significance. The statistical analysis was performed using IBM SPSS Statistic software (v.21) and R software (v. 4.0.1) [29]. R statistic packages used included compareGroups [30], car [31], ggcorrplot [32], and corrplot [33].

## 3. Results

The main sociodemographic and clinical characteristics are summarized in Table 1. As expected, a female predominance was observed, accounting for 91% of the sample. Patients were mainly inactive, receiving low-dose prednisone and universal antimalarial therapy, in line with the therapeutic schedules of the Lupus–Cruces cohort [18]. Most patients were in remission, as depicted by a mean (SD) SLEDAI-2K score of 1.59 (2.44). Despite a median disease duration longer than 10 years, the degree of damage accrual was low, with a mean (SD) SDI of 0.33 (0.84), similar to what has been previously reported in our cohort [18]. No significant differences were found in any of the autoimmune clinical manifestations or analytical parameters between the groups (Table 1).

Altogether, the psychopathological assessment revealed that 23 patients (25%) presented a heterogeneous group of clinical syndromes that met the clinical criteria for a psychiatric disorder (Table 2). Additionally, according to the main clinical guidelines, the psychiatric evaluation revealed that nine patients (9.8%) had psychosocial disturbances that could not be classified as mental disorders. In this group of patients, primary support group-related problems and difficulties in managing daily life circumstances were among the main psychosocial difficulties reported.

Among patients who met the diagnostic criteria for a current major psychiatric diagnosis, 15 (16.3% of the cohort) met the ICD-10 criteria for a depressive mood disorder, which was in fact the most common prevalent disorder. According to ICD-10 criteria, only one patient had an in remission organic mood disorder diagnosis.

In contrast, when using the scores obtained on the main clinical scales for the assessment of depression exclusively as diagnostic criteria, and according to the cut-off criteria of the MADRS, as many as 25 patients (27%) met the criteria for depression. Among them, 20 patients (21.7%) met the symptom criteria for “mild depression”, 4 patients (4.3%) had “moderate depression”, and 1 patient (1.1%) presented “severe depression”.

### 3.1. Autoimmune Clinical Predictors of Presenting a Mood Disorder

Regarding their main clinical characteristics, patients with and without a current mood disorder did not differ significantly in the age at inclusion (*p* = 0.995) or in disease duration (*p* = 0.227). Moreover, as observed in Table 1, patients did not differ on the main clinical variables. No differences were observed among the groups regarding the SLEDAI-2K (*p* = 0.995) and SDI scores (*p* = 0.926); there were no significant differences in current treatment with hydroxychloroquine (*p* = 0.16); immunosuppressive drugs (*p* = 0.691); or in the dose of prednisone, either current (*p* = 0.55) or cumulative (*p* = 0.691). No significant correlations were found between the severity of depression, as measured by the MADRS and the SLEDAI-2K (rho = –0.111, *p* = 0.292) or the SDI scores (rho = –0.043, *p* = 0.681).

### 3.2. Psychiatric Predictors of Mood Disorder in SLE Patients

Regarding psychopathological assessment, there were significant differences between the groups (*p* < 0.001). Overall, patients with SLE with comorbid major disorder presented a greater percentage of family psychiatric antecedents (*p* = 0.003). Likewise, higher anxiety scores (*p* < 0.001) increased the YMRS and the Plutchik Suicide Risk Scale scores (*p* < 0.001) in SLE patients presenting with a mood disorder. Similarly, greater clinical severity, as measured by the CGI scale, was observed in SLE patients with mood disorders, and a trend toward greater exposure to psychological distress due to childhood traumatic experiences was found.

In the univariate regression, age, ethnicity, the MADRS total score, the HARS total score, the Plutchick Suicide Risk Scale scores, the YMRS scores, the CGI scores, and the reported psychiatric family history had significant associations with presenting a mood disorder. Before adjustment in the multivariate model, ethnicity was removed because its values were not representative. Similarly, the HARS was also removed because it presented a correlation coefficient of 0.83 with MADRS and was related to anxiety, not depression. The remaining variables presented correlation coefficients ≤ 0.60 and appropriate VIF values (<5). As a result, these factors were included as independent variables in the multiple logistic regression model.

**Table 1:** Sociodemographic and clinical characteristics of the sample included in the analysis.

	Total Sample (*n* = 92)	Patients without a Mood Disorder (*n* = 77)	Patients with Mood Disorders (*n* = 15)	N	*p* Value
**Gender**—female	84 (91.3%)	71 (92.2%)	13 (86.7%)	92	0.612
**Ethnicity** Caucasian Hispanic Arabic	86 (93.5%) 5 (5.4%) 1 (1.1%)	74 (96.1%) 2 (2.6%) 1 (1.3%)	12 (80%) 3 (20%) -	92	0.053
**Age at inclusion**, *Mean (SD)*:	44.04 (11.87)	43.23 (11.89)	48.20 (11.23)	92	0.136
**Disease duration**, *Median [25th;75th]*:	11.00 [6.00;18.00]	11.00 [6.00;18.00]	9.00 [7.00;21.50]	92	0.966
**Main clinical manifestations** Articular Cutaneous Serosal Hematological Renal Antiphospholipid syndrome	65 (71.4%) 46 (50.5%) 17 (18.7%) 11 (12.1%) 19 (20.9%) 8 (8.7%)	54 (21.1%) 40 (52.6%) 15 (19.7%) 10 (13.2%) 14 (18.4%) 8 (10.4%)	11 (73.3%) 6 (40%) 2 (13.3%) 1 (6.7%) 5 (33.3%) 0 (0%)	92	1.000 0.410 0.728 0.684 0.294 0.345
**SLEDAI-2K**, *Mean (SD)*:	1.59 (2.44)	1.64 (2.60)	1.31 (1.49)	92	0.995
**SDI**, *Mean (SD)*:	0.33 (0.84)	0.32 (0.84)	0.38 (0.89)	92	0.802
**Positive anti-dsDNA**	25 (27.2%)	19 (24.7%)	6 (40%)	92	0.224
**C3 (mg/dL)**, *Mean (SD)*:	99.34 (21.31)	98.29 (20.83)	105.07 (25.50)	92	0.397
**C4 (mg/dL)**, *Mean (SD)*:	20.38 (8.13)	20.33 (8.24)	20.64 (7.78)	92	0.721
**Anti-ribosomal *p* positive**, *N (%)*	9 (9.8%)	7 (9.1%)	2 (13.3%)	92	0.637
**Prednisone dose (mg/day)**, *Mean (SD)*:	1.94 (1.94)	1.92 (1.97)	2.03 (1.88)	92	0.553
**Cumulative prednisone dose in 1 year (mg)**, *Mean (SD)*:	671.12 (615.34)	659.61 (626.47)	725.78 (575.24)	92	0.513
**Hydroxychloroquine drug therapy (yes)**, *N (%)*:	90 (97.8%)	77 (100%)	14 (93.3%)	92	0.163
**Other immunosuppressive drug therapy (yes)**, *N (%)*:	34 (36.96%)	26 (33.77%)	8 (53.33%)	92	0.253
**Psychiatric family history (yes)**, *N (%)*	50 (54.95%)	36 (47.37%)	14 (93.33%)	92	**0.003**
**Hamilton Anxiety Rating Scale (HARS)**, *Median [25th;75th]*:	2.00 [1.00;6.00]	1.00 [0.00;4.00]	9.00 [6.00;18.50]	92	**<0.001**
**Montgomery–Åsberg Depression Rating Scale (MADRS)**, *Median [25th;75th]*:	2.00 [0.00;7.00]	1.00 [0.00;4.00]	12.00 [8.50;22.50]	92	**<0.001**
**Young Mania Rating Scale (YMRS)**, *Median [25th;75th]*:	0.00 [0.00;0.00]	0.00 [0.00;0.00]	0.00 [0.00;3.00]	92	**<0.001**
**Plutchik Suicide Risk Scale**, *Median [25th;75th]*:	2.00 [1.00;4.25]	1.00 [1.00;3.00]	5.00 [3.00;7.00]	92	**<0.001**
**Global Clinical Impression (CGI)**, *N (%)*: 1. Normal, not ill	65 (70.65%)	64 (83.12%)	1 (7.14%)	91	**<0.001**
2. Borderline mental ill	8 (8.70%)	6 (7.79%)	2 (14.29%%)		
3. Mildly ill	12 (13.19%)	5 (6.49%)	7 (50.00%)		
4. Moderately ill	5 (5.43%)	2 (2.60%)	3 (21.43%)		
5. Markedly ill	1 (1.09%)	0 (0.00%)	1 (7.14%)		
**Traumatic Experiences Screening Questionnaire (ExpTra-S)**, *Median [25th;75th]* Frequency	0.00 [0.00;2.00]	0.00 [0.00;2.00]	1.00 [0.50;6.00]	87	**0.031**
Distress	0.00 [0.00;3.00]	0.00 [0.00;2.00]	3.00 [1.00;6.00]	87	**0.006**

**SD**: standard deviation; **SLEDAI-2K:** Systemic Lupus Erythematosus Disease Activity Index; **SDI:** Systemic Lupus International Collaborating Clinics/ACR Damage Index.

**Table 2:** Prevalence of mental disorders.

Current Psychiatric Diagnoses According to ICD-10 (*n* = 32)	N (%)
**Mood disorders** Major depressive disorder Persistent depressive disorder **Trauma and Stress-Related Disorders** Adjustment disorder **Other disorders** Organic depressive disorder—*in remission* Generalized anxiety disorder Eating disorder **Psychosocial conditions not attributable to a mental disorder** Code Z63. Problems related to primary support group Code Z73. Problems related to life management difficulty	13 (40.6%) 2 (6.3%) 5 (15.6%) 1 (3.1%) 1 (3.1%) 1 (3.1%) 4 (12.5%) 5 (15.6%)

**Figure 1:**
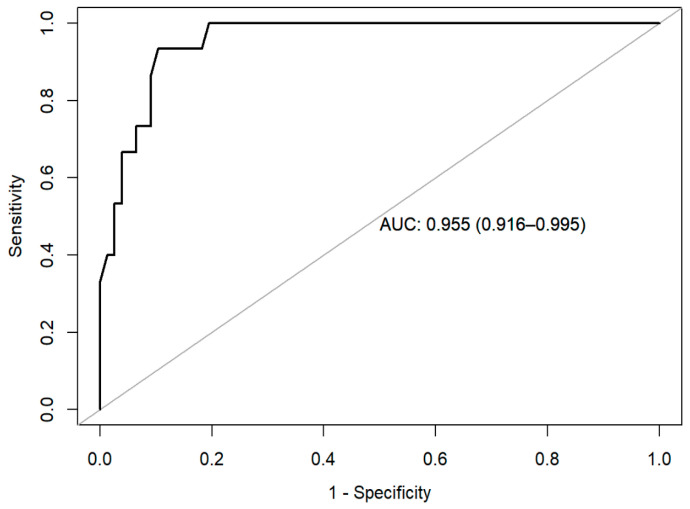
ROC curve. The figure shows the area under the curve (AUC) of the receiver operating characteristic (ROC).

As shown in Table 3, the final model revealed a statistically significant association between presenting a mood disorder, the MADRS total scores, and the YMRS total scores (*p* < 0.001). The Hosmer–Lemeshow statistic was 6.398 (*p* = 0.603), suggesting a well-calibrated predictive model. These findings suggest that having a mood disorder is associated with greater levels of depressive symptoms and psychiatric disease severity. We further estimated the prediction efficacy of the model by performing an AUC analysis, showing a value of 0.965 (Figure 1). Altogether, these results indicate that the model can precisely detect the presence of a mood disorder in SLE patients.

## 4. Discussion

The aim of this study was to assess the real prevalence of well-characterized mood disorders in a well-defined cohort of SLE patients. Based on our results, patients with SLE showed lower prevalence rates of depressive disorders than those previously reported in the literature. In line with this finding, the prevalence of other psychopathological disturbances, such as anxiety disorders, was also low, with adjustment disorder being the second most common diagnosis. Furthermore, no SLE-related factors appear to influence the presentation of a mood disorder, with psychiatric clinical factors having a significant effect on SLE-depressed patients.

Our findings contrast with previous studies that reported a higher prevalence of psychiatric disorders among patients with lupus [34,35,36,37]. Although some studies reported prevalence rates similar to those obtained in our cohort, non-standardized scales were used to support the clinical diagnosis [38]. However, despite this lower prevalence compared to other SLE cohorts, we observed a prevalence of depressive disorders higher than in the general population. According to The Global Health Data Exchange, the overall prevalence of depressive disorders in Spain is 4.13% [39], with variations depending on the methodological aspects of the individual studies [16]. Therefore, it is essential to assess the presence of psychological disturbances in patients with autoimmune diseases through a thorough psychiatric evaluation. Since NPSLE manifestations have a negative impact on the quality of life and day-to-day functional outcomes, the early detection of mental disorders may lead to global improvement in SLE patients [40].

A significant proportion of patients had psychological disturbances that could not be classified as a mental disorder. This classification encompasses a broad spectrum of nonspecific major psychiatric disturbances, which influence health status and attendance to health services. In fact, this reflects the importance of sociocultural circumstances in the emergence and persistence of psychological disturbances. The successful identification and management of these disorders facilitate the adaption of therapeutic interventions based on the individual characteristics of each patient.

It is worth noting that, based on the cut-off criteria of the scores obtained on the MADRS to assess the occurrence of depressive symptoms, approximately 27 percent of our study group would have depression. This fact emphasizes the need for a thorough psychiatric evaluation, which can be supported by clinical screening tools such as the MADRS, among others, for the diagnosis of mental disorders.

We found no correlation between the presence of psychiatric disorders and any of the main autoimmune clinical characteristics of SLE, such as disease duration, disease activity, cumulative disease damage, or immunosuppressive treatment. It is important to note the low prednisone dose and the high use of hydroxychloroquine in both subgroups. The low degree of accrued damage in our cohort has a definite relation with the therapeutic schemes used in our unit [41].

On the other hand, the main psychiatric scales assessed revealed significant differences between groups. Among the main clinical correlators predicting the presentation of a mood disorder, we found global scores such as the MADRS, used to assess depression severity, and the total scores of the Young Mania Rating Scale. Besides that, and despite not being included in the final regression model, the higher overall anxiety scores, the higher scores observed in the Plutchick Risk Suicide Scale, and the greater presence of early traumatic experiences are of clinical significance. On this matter, the impact of early traumatic experiences on the immune system functioning is well established [42,43]. Major pro-inflammatory factors, including IFN-y, Interlucin-6, and TNF-α, among others, have been found to be increased [42]. While research on early traumatic experiences has been replicated in a variety of psychiatric populations, few studies on autoimmune disorders have taken this factor into account.

The main limitations of this study include its limited sample size, particularly in terms of the percentage of SLE patients with mood disorders. Similarly, not all patients in the cohort were evaluated, as only patients who voluntarily agreed to participate in the study were included, all of them in an outpatient clinic setting. Thus, it is possible that some patients presenting with mood disorders have not been effectively assessed. In addition, the large number of patients on hydroxychloroquine and the low current and cumulative dose of prednisone, as well as the low level of activity and damage accrual, as has been shown in our patients in previous studies [41], could have hampered the analysis of the possible predictors of mood disorders. On the other hand, the role of immunosuppressive therapy other than glucocorticoids in the development or protection against mood disorders in SLE patients has not been established [38]. Overall, the availability of a multidisciplinary team for the management of patients with autoimmune diseases should be highlighted as a possible additional explanation for our findings. Patients who show early signs of psychological problems are evaluated jointly by an autoimmune disease team psychiatrist. In this regard, close clinical care and follow-up by a multidisciplinary team can benefit from the early detection of emotional disturbances and prompt rapid therapeutic interventions. This includes a correct diagnosis with the use of a well-accepted and widely standardized semi-structured psychiatric assessment instrument (SCID), which was used in this study in accordance with the ACR guidelines for the assessment of NPSLE [13].

## 5. Conclusions

In summary, the prevalence of mood disorders in SLE using a comprehensive standardized interview conducted by a trained mental health specialist was lower than previously reported. No associations were found with SLE clinical features, disease activity, damage, or specific therapies. Our results highlight the need for multidisciplinary teamwork for the early diagnosis and therapeutic management of SLE patients with neuropsychiatric disorders.

## Figures and Tables

**Table 3:** Multiple logistic regression analysis of predictors for mood disorders in SLE patients.

Variables	OR	95% CI	*p*-Value
MADRS total score	1.373	1.180 to 1.679	<0.001
YMRS total score	3.009	1.202 to 10.56	0.022

**Abbreviations: OR** = odds ratio; **CI** = confidence interval; **MADRS** = Montgomery–Åsberg Depression Rating Scale, **YMRS** = Young Mania Rating Scale.

## Data Availability

The data that support the findings of this study are available upon request from the corresponding author. The data are not publicly available due to privacy or ethical restrictions.
